# Mitigating the Effects of a Pandemic: Facilitating Improved Nursing Home Care Delivery Through Technology

**DOI:** 10.2196/20110

**Published:** 2020-05-26

**Authors:** Linda S Edelman, Eleanor S McConnell, Susan M Kennerly, Jenny Alderden, Susan D Horn, Tracey L Yap

**Affiliations:** 1 College of Nursing University of Utah Salt Lake City, UT United States; 2 School of Nursing Duke University Durham, NC United States; 3 Geriatric Research, Education and Clinical Center (GRECC) Durham VA Healthcare System Department of Veterans Affairs Durham, NC United States; 4 College of Nursing East Carolina University Greenville, NC United States; 5 Department of Population Health Sciences School of Medicine University of Utah Salt Lake City, UT United States

**Keywords:** nursing home, technology, social isolation, covid-19

## Abstract

The coronavirus disease (COVID-19) pandemic has been particularly challenging for nursing home staff and residents. Centers for Medicare & Medicaid Services regulation waivers are burdening staff and affecting how care is delivered. Residents are experiencing social isolation, which can result in physical and behavioral health issues, particularly for persons with dementia. These challenges can be addressed in part through technology adaptations. Full integration of electronic health record systems can improve workflow and care quality. Telehealth can improve access to outside providers, provide remote monitoring, and improve social connectedness. Electronic and audiovisual programs can be used for end-of-life planning and information sharing between nursing home staff and families. Online learning systems and other online resources provide flexible options for staff education and training. Investing in and adapting technology can help mitigate workforce stress and improve the quality of nursing home care during and after the COVID-19 crisis.

## Introduction

The coronavirus disease (COVID-19) pandemic has placed unprecedented strain on the US health care system, and the possibility that it could become endemic—meaning it will be a common dweller that regularly infects humans—suggests that this strain will persist for some time. As COVID-19 cases accelerate, health care workers are becoming more vital than ever, and they are rightly being hailed as heroes for their generous, brave dedication to caring for patients at the risk of their own health. Providing these heroes with resources to safely deliver high-quality care to their patients and themselves is imperative not only in acute care settings such as emergency rooms and intensive care units (ICUs), but also in nursing homes, where staff provide care for vulnerable older adults in settings that are at risk for infectious disease outbreaks. Nursing home staff face unique challenges during the COVID-19 pandemic because they care for older adults with a high level of vulnerability to COVID-19 and experience constraints that far exceed those imposed in acute care settings. Telehealth strategies, automatic clinical decision-making reports, and other uses of technology commonly found in other care settings hold great promise to improve nursing home care and outcomes.

By 2030, almost 1 in 5 Americans will be 65 years or older [1], and today, >1.3 million people live in 15,600 nursing homes [2]; in society, these people are some of the most frail and vulnerable to viral infections such as COVID-19 because they are older, have other medical conditions such as heart and respiratory disease, and live in group settings. US nursing homes are familiar with disease outbreaks, including seasonal influenza and norovirus; although COVID-19 shares similarities with these outbreaks, it will be more devastating because there are no approved therapeutics to slow the course of its toll on the human body and because there is currently no vaccine available to combat it.

Because of these vulnerabilities, nursing homes were early reporters of positive COVID-19 cases. The first reported COVID-19 death in the United States was a nursing home resident in Kirkland, Washington, reported on February 28, 2020. Nursing homes across the country and the Centers for Medicare and Medicaid Services (CMS) acted quickly; by March 13, 2020, they enacted guidelines to protect nursing home residents, including barring visitors and adding infection control measures that restrict communal activities, essentially isolating residents in their rooms. Despite these measures, COVID-19 infections spread quickly between nursing home residents and staff. As of April 29, 2020, more than 2700 Medicare-certified nursing homes across the country (1 in 6 facilities) acknowledged infections in residents or staff, and in some states, a majority of nursing homes have reported COVID-19 cases [3]. Nationally, COVID-19 death rates are higher for nursing home residents, who account for over half of deaths in some states [4].

Currently, nursing homes continue to battle COVID-19 infections among residents and staff, which is challenging how nursing homes provide care to our most vulnerable older adults. In addition to preventing the spread of COVID-19 within nursing homes, it is now necessary to plan for the admission of patients who were hospitalized with COVID-19 and are too debilitated to return to their homes. The recovery trajectories and continued health care needs of these patients are uncharted; however, increasing evidence points to long-term post–COVID-19 complications that may increase the complexity and length of rehabilitation [5].

During the COVID-19 pandemic, there has been much focus on personal protective equipment (PPE), the lack of testing and supplies, and the need for infection control training; however, this pandemic has also highlighted problems that nursing homes were already facing, such as frequent staff shortages and high turnover. It is important to take this opportunity to increase awareness of other aspects of care delivery within nursing homes that have been affected by the pandemic. These aspects of care delivery can be enhanced by leveraging strategies such as technology used in other areas of the healthcare system to improve nursing home care both now and in the future.

## What challenges and issues do nursing homes face?

COVID-19 has presented unique challenges and highlighted ongoing issues faced by nursing homes in providing effective, compassionate, and safe care for vulnerable older adults in an institutional home setting. While the strict regulatory controls currently in place for nursing homes have likely prevented further spread of COVID-19 and subsequent deaths, nursing home staff are being challenged with new work patterns, longer hours, and the need to find new ways to communicate with families. These measures pose additional risks for nursing home residents, such as isolation, which limits their mobility and social interactions. Furthermore, family members are not able to visit their loved ones, which can be particularly devastating when residents have dementia or are at end-of-life. Together, residents, families, and nursing home staff are facing unprecedented stress as they navigate these challenges and their own fears about the virus.

### Impact of CMS Regulations on Care Delivery

Prior to COVID-19, the nursing home industry was highly regulated by CMS, which partners with state survey agencies to monitor every Medicare-certified and Medicaid-certified nursing home for safety and quality. Many of these challenges are related to CMS regulations on how nursing home care is delivered.

#### Regulation Waivers

During the COVID-19 pandemic, CMS has provided nursing homes with flexibility to decrease COVID-19 infection risk, emphasizing resident care over paperwork [6]. These waivers impact physical building requirements and relax minimum data set and staffing data submissions. Resident admission and discharge planning requirements are being waived. Quality assurance and performance improvement (QAPI) requirements are now focused on adverse events and infection control. Certified nursing assistant (CNA) certifications and annual in-service training requirements are being waived. Physicians can delegate any tasks and visits to physician assistants, nurse practitioners, or clinical nurse specialists who are acting within state scope of practice laws. Telehealth regulations have been relaxed, and billable physician visits can be conducted via telehealth, including telephone visits. Staying abreast of evolving CMS, Centers for Disease Control and Prevention (CDC), and state regulations and guidelines is challenging for an already stressed workforce. In particular, implementing new and difficult infection control guidelines, such as stopping congregant activities and preventing families from visiting their loved ones, is particularly stressful for staff, who recognize the impact on residents’ general health and quality of life.

#### Staffing and Workforce Issues

Nursing home staff, including nurses, physical and occupational therapists, social workers, and direct care workers, often work at several different facilities; this increases the risk of acquiring and spreading COVID-19 between facilities, especially since many people who are positive for COVID-19 do not show outward symptoms. As the virus spreads among staff and residents, large numbers of nursing home personnel are remaining at home, contributing further to often pre-existing staff shortages. Some nursing homes must rely on agency staff at the risk of inconsistent care delivery and documentation. The relaxed CMS regulations support the hiring of new CNAs to fill vacancies; however, due to the lack of training requirements, more staff are unprepared to work with nursing home residents during the pandemic. Further, staff must adjust to workflow changes resulting from residents being confined to their rooms and the added time required for implementing infection control practices. The negative impact of social isolation on the physical and mental health of residents further contributes to staff stress, particularly in the face of the probable endemic nature of COVID-19.

#### Infection Control Constraints

Nursing homes must ensure that they are adhering to infection control guidelines issued by a number of CDC, CMS, state, and local regulatory bodies [7]. New guidance and regulations are announced frequently and often appear to contradict those of other agencies. Every individual entering a nursing home must be screened for COVID-19 symptoms, and every resident must have their temperature checked daily [8]. Staff must wear masks the entire time they are in the nursing home, and if there are residents with COVID-19, full PPE must be worn when working with any resident [7]. Residents should wear masks when any staff member is in their room. These measures increase the time and difficulty of working with residents, increasing the difficulty of person-centered care and increasing the stress on staff and residents alike.

### Impact on Residents

Under COVID-19 restrictions, nursing homes are closed to everyone but essential health care providers and staff. Congregant activities, including meals, are not allowed, and residents are mainly confined to their rooms. The consequences of isolation on the physical and mental health of nursing home residents will not be known for some time. However, anecdotal reports from staff and families suggest that residents’ health is declining rapidly as a result of COVID-19 isolation. Below, we discuss several areas of particular concern.

#### Social Isolation

Because COVID-19 regulations prevent outside visitors and congregating of nursing home residents, the residents are socially isolated from each other and from family and friends. Social isolation itself is associated with increased loneliness, which has been associated with a plethora of behavioral and physical health issues, including increased depression and anxiety, increased risk of falls and hospitalization, and even death [9]. Residents living with dementia face particular challenges, as they often cannot understand why their routines are being disrupted and their activities curtailed. These disruptions, in addition to the lack of visitors who are often able to calm cognitively impaired residents, can result in a host of behavioral symptoms that staff have less time to address. Short-term consequences of social isolation are being reported anecdotally from health care providers, nursing home staff, and family members; these consequences include rapid decline in function and health (eg, dehydration, renal problems, and malnutrition), hopelessness and severe depression, and increases in suicidal ideation [10].

#### Increased Pressure Injury Risk

Nursing home residents are at increased risk for pressure injuries because they already have limited mobility and are now being confined to their rooms. Residents will spend more time sitting or lying down; this increases the intensity and duration of pressure exposure, which are two factors that lead to pressure injury development [11]. The use of prone positioning in COVID-19 treatment further increases risk of pressure injuries, particularly on the face [12].

#### Increased Hospitalization Risk

As residents spend more time in their rooms, they are at risk for physical deconditioning, malnutrition (due to a lack of shared meals or assistance with eating), and depressive symptoms, all of which increase risk of hospitalization [13,14]. Dehydration and its associated risk of urinary tract infection and renal problems is also a concern due to the lack of shared meals and, potentially, to decreases in the amount of time staff have available for offering and encouraging oral hydration [15].

## How can technology be leveraged to improve the care nurses deliver and keep nurses and residents safe?

### Electronic Health Records

Electronic health record systems have been used in other health care settings to improve workflow and quality of care; however, full integration has been slower in nursing home settings. Enabling all nursing home staff to access and document records electronically provides an opportunity for real-time communication between staff members. However, many nursing homes only have computers at central nursing stations that are distant from resident rooms. For electronic charting to be efficient, adaptation of mobile charting platforms is needed, such as the use of tablets or point-of-care mobile workstations. The pandemic has also greatly stimulated the need to increase use of electronic medical record systems to collect data and use them for clinical decision-making in nursing homes. Several potential technology solutions are proposed below.

### Standardized Documentation and Real-Time (On-Time) Reports to Improve Quality of Care

One example of successful implementation of health information technology is the On-Time Quality Improvement for Long Term Care (On-Time) program to decrease high pressure ulcer incidence rates in nursing homes [16-19]. Working with front-line staff, including CNAs, nurses, and dietary staff, project facilitators evaluated, streamlined, and designed standardized CNA documentation that incorporated best practice elements into daily charting. The goal was to facilitate consistently good preventive care using the daily information nurses need to target resources to residents at risk of developing pressure ulcers. The developed tools included a documentation form, a documentation completeness report, and four additional weekly clinical decision-making reports that helped identify residents at risk.

Development and testing were followed by incorporation of the On-Time tools into more than 10 different long-term care electronic medical record systems. Using electronic medical records to implement On-Time standardized documentation and clinical decision-making reports in nursing homes resulted in enhanced quality improvement (QI) efforts; focusing staff on high-risk residents, improving team communication, and prompting timely interventions; providing a clear and practical process to maximize the role and contribution of CNAs in pressure ulcer prevention; and increasing CNA engagement with QI by showing them how their documentation, summarized in clinical reports, is used as a basis for proactive clinical decision-making. An evaluation of the On-Time Pressure Ulcer Prevention Program in 12 New York State nursing homes (3463 residents) found a large and statistically significant reduction in pressure ulcer incidence associated with the joint implementation of the four core On-Time reports [20-22].

### Telehealth

Telehealth encompasses a broad range of electronic information and telecommunication technologies to support long-distance clinical health care and related activities through videoconferencing, internet-based applications, store-and-forward imaging, streaming media, and telephone-based services [23]. Telehealth approaches to improve care access and outcomes among older adults have been extensively studied and are associated with high degrees of patient and caregiver satisfaction [24]. Nursing home professional organizations such as LeadingAge have developed guidance regarding how to evaluate telehealth applications for use in long-term care [25]. Enhanced use of telehealth holds great potential to reduce burden on nursing home staff, enhance integration of specialty expertise care of nursing home residents, and enrich connections with family and friends, enhancing social connection during required physical distancing. For example, in acute care settings, use of tablets or telepresence robots has allowed staff to interact more with patients while reducing the duration of contact and use of PPE [26]; these devices have also been found to be acceptable to older adults [27]. Likewise, telehealth interventions connecting cardiologists to skilled nursing facility residents permit more frequent monitoring without direct visits and have been shown to reduce rehospitalization following heart failure [28].

Nursing homes should consider adopting telehealth approaches that have been successfully used in other health care settings to help keep nursing home residents safe and facilitate social connection while observing physical distancing. The Electronic Intensive Care Unit (eICU) uses two-way cameras and video monitors connected to a central hub for remote monitoring and care delivery [29]. Although this technology is primarily used for monitoring physiological processes, eICU staff also provide social support and “rounds” for patients to identify any unmet needs, which they communicate to ICU nurses. A similar process could be implemented in the nursing home setting, where video-enabled rounds, especially for residents who have tested positive for COVID-19, would allow staff to avoid donning and doffing PPE and enhance nursing home residents’ ability to express their needs and to experience regular human contact, albeit virtually. Video contact is particularly beneficial for residents with hearing impairment who rely on visual cues and mouth movements that are obstructed by PPE such as masks. Video-enabled rounding could also be used during mealtimes for residents who require prompting to eat and drink or for residents who simply desire company during meals.

Several barriers to expanded use of telehealth in nursing homes merit attention. First, older adults may be less familiar with tablets or mobile phones and may require initial face-to-face orientation to a new platform [30]. Use of tablets with “one-touch” connection features that permit adjustments of font size or volume and accommodate the use of device holders to compensate for arthritis or upper extremity weakness are recommended [31].

### Using Technology in End-of-Life Care Planning

Now more than ever, information sharing between nursing home providers and staff, residents, and family members is particularly important at end-of-life to ensure resident preferences are respected. Since 2016, Medicare has reimbursed providers for formalized advance care planning discussions that can be conducted by telehealth visits [32]. Advance directives and other end-of-life documentation, such as the POLST (Physician Orders for Life-Sustaining Treatment), are useful in helping families and surrogates make decisions that are in keeping with the wishes of the dying resident and can guide the actions of nursing home staff. However, family members are often unaware of what was documented. Recent research has demonstrated that technology can be used to bridge this gap. An example is the use of Me & My Wishes videos of residents’ preferences for daily and end-of-life care that can be shared with designated family members and nursing home staff [33]. While filming and editing videos of nursing home residents discussing end-of-life preferences is likely not feasible during the COVID-19 pandemic, real-time discussions between residents, family members, and providers using tablet or smartphone technology are possible and would help ensure that care preferences are met. iPads are facilitating end-of-life conversations between hospitalized COVID-19 patients and family members [34] and could be used similarly in nursing homes both for decision-making and to allow families to say goodbye to loved ones.

### Access to Technology to Promote Social Interaction

In addition to telehealth technologies, videoconferencing and tablet-based applications have been used to enhance social interaction among nursing home residents and family [35]. However, wide adoption has been impeded by limited wireless access within facilities as well as by financial limitations on access to devices [36]. ICU diaries, many of which are free or inexpensive, may have a role in the nursing home setting to facilitate communication during periods of physical/social distancing. These diaries are routinely used in hospitals to assimilate care information and to allow families and friends to maintain a connection, resulting in decreased anxiety and depression among ICU survivors and less posttraumatic stress in survivors’ families [37]. In addition to clinical information, ICU diaries include details about events occurring at home and in the community, enabling patients and their families to stay abreast of each other’s lives during periods of social distancing [38]. To make the above technologies available to residents who have mobility and sensory impairments, simple “hacks” to telemedicine equipment, such as placing monitors on flexible stands so that they can be moved in front of residents regardless of whether they are sitting, reclining, or lying in bed, could keep this technology in reach of residents. Devices such as Apple TV can enlarge smartphone or tablet screens for easier viewing. Ensuring that hearing aids and eyeglasses are available should enhance older adults’ ability to communicate with a wider array of friends and family.

### Technology to Support Staff Education and Training

Relaxed guidelines for CNA training, the need to train new or agency staff quickly, and the increased workloads faced by nursing home staff increase the need for staff education and training. Technology can be leveraged to address these challenges and to avoid bringing staff together for training. Some nursing homes are already using learning management systems for staff training. Other online educational modules and training videos from reputable sources such as the CDC, health systems, and professional organizations can also be used. For example, training on donning and doffing PPE is needed, and demonstration videos that provide this training are available from a number of sources. The CDC recommends that nursing homes have a staff member available for every shift to monitor PPE use; this staff member could assess and approve staff members’ PPE use. Almost all of these online training programs can be accessed on a smartphone or tablet, which can help staff complete required training.

## Conclusion

The unprecedented challenges of the COVID-19 pandemic place particularly great burdens on nursing home staff who are unfairly stigmatized and had the fewest resources in prepandemic times. At the same time, the dedication and sacrifice of the majority of nursing home staff go unnoticed. Today, more than ever, it is important that we support nursing home staff, particularly those providing direct care such as nursing, dietary, social, therapy, pharmacy, and custodial services, to our most vulnerable older adults in their time of greatest need. Technology presents opportunities to address the challenges these staff members are currently facing. We owe increased support to vulnerable older people and those who care for them, and we call for an investment in technology and other resources to support older people and their caregivers during the pandemic.

## Abbreviations

CDC: Centers for Disease Control and Prevention
CMS: Centers for Medicare & Medicaid Services
CNA: certified nursing assistant
COVID-19: coronavirus disease
eICU: electronic intensive care unit
ICU: intensive care unit
POLST: Physician Orders for Life-Sustaining Treatment
PPE: personal protective equipment
QAPI: quality assurance and performance improvement
QI: quality improvement
